# Levodopa Impairs Lysosomal Function in Sensory Neurons In Vitro

**DOI:** 10.3390/biology13110893

**Published:** 2024-11-02

**Authors:** Oyedele J. Olaoye, Asya Esin Aksoy, Santeri V. Hyytiäinen, Aia A. Narits, Miriam A. Hickey

**Affiliations:** Department of Pharmacology, Institute of Biomedicine and Translational Medicine, University of Tartu, 50411 Tartu, Estonia; oyedele.olaoye@auckland.ac.nz (O.J.O.); asya.esin.aksoy@ut.ee (A.E.A.); santeri.veini.hyytiainen@ut.ee (S.V.H.); aiaadele@ut.ee (A.A.N.)

**Keywords:** dorsal root ganglion, rotenone, levodopa, mitochondria, lysosome, Parkinson’s disease, 50B11 sensory cell line

## Abstract

Parkinson’s disease (PD) is the second-most common neurodegenerative disease worldwide. Patients are diagnosed based upon movement disorders, including bradykinesia, tremor and stiffness of movement. However, non-motor signs, including constipation, rapid eye movement sleep behavior disorder, smell deficits and pain are well recognized. Peripheral neuropathy is also increasingly recognized, as the vast majority of patients show reduced intraepidermal nerve fibers, and sensory nerve conduction and sensory function is also impaired. Many case studies in the literature show that high-dose levodopa may induce or exacerbate neuropathy in PD, which is thought to involve levodopa’s metabolism to homocysteine. Here, we treated primary cultures of dorsal root ganglia and a sensory neuronal cell line with levodopa to examine effects on cell morphology, mitochondrial content and physiology, and lysosomal function. High-dose levodopa reduced mitochondrial membrane potential. At concentrations observed in the patient, levodopa enhanced immunoreactivity to beta III tubulin. Critically, levodopa reduced lysosomal content and also reduced the proportion of lysosomes that were acidic, thereby impairing their function, whereas homocysteine tended to increase lysosome content. Levodopa is a critically important drug for the treatment of PD. However, our data suggest that at concentrations observed in the patient, it has deleterious effects on sensory neurons that are not related to homocysteine.

## Simple Summary

Parkinson’s disease (PD) is one of the most common chronic, degenerative brain diseases worldwide. Patients are diagnosed on the basis of slowness of movement and/or tremor and/or stiffness. However, many symptoms that are not movement related are now well recognized. Patients show changes in skin sensation, and the vast majority of patients show loss of sensory neurites, which enable sensation in skin. These changes in skin sensation occur prior to diagnosis; however, sensory issues may also be exacerbated by levodopa, an important drug used in the treatment of PD. Undoubtedly, levodopa is critical for the treatment of PD, but at high doses, it has repeatedly been shown to impair sensation in PD patients. Here, we show for the first time that high-dose levodopa impairs function of sensory neurons. Importantly, we also show for the first time that lysosomes, a critical organelle involved in recycling, are impaired by levodopa concentrations observed in patients. These data are important given the well-known lysosomal dysfunction observed in PD. Our data shed light on how levodopa, the most important drug in the treatment of PD, may contribute to sensory deficits in PD.

## 1. Introduction

Parkinson’s disease (PD) is the most prevalent neurodegenerative motor disorder worldwide, and indeed, it is currently placed 11th in the global burden of disorders affecting the nervous system, with DALYS (disability-adjusted life years) increased by 10% since 1990 [1]. It was only in 1960 that Hornykiewicz first published the loss of dopamine in the striatum of patients with PD [2]. A prior study in the 1930s had shown that dopa decarboxylase converted levodopa to dopamine [3], and Hornykiewicz and colleagues very quickly moved forward to a clinical trial of levodopa to show beneficial effects of levodopa that were not seen with related molecules [4,5]. Levodopa, as a chronic oral drug for PD, was established by 1967 [6], fast becoming the mainstay of treatment and the treatment of choice among patients [7] and neurologists [8,9,10]. Undoubtedly, levodopa treatment of PD is a success story.

The diagnostic movement disorder of PD is largely due to the loss of dopamine neurons of the substantia nigra pars compacta (SNpc), a tiny nucleus in midbrain with a critical projection to striatum. Although the precise mechanism(s) underlying loss of motor control in PD remains unclear, this loss of neurons removes a modulating input to striatal output neurons. Loss of dopaminergic neurons of the SNpc in animals also leads to movement disorders [11], which are well treated with levodopa [12,13], and some of the movement disorders of PD are reproduced pharmacologically in schizophrenic patients treated with dopamine antagonists [14]. The specific cause of cell death in PD also remains unclear, although risk factors for PD include traumatic brain injury [15] and pesticides such as rotenone and paraquat [16], which are complex I inhibitors that lead to oxidative stress and mitochondrial impairment [17]. Genetic causes of PD are rare but very informative and include mutations and multiplications of the *SNCA* gene, which encodes alpha synuclein [18]. In aggregated form, alpha synuclein is the majority protein in the Lewy body, a hallmark of PD neuropathology [19]. This protein is normally located in the vertebrate presynaptic nerve terminal, with a weak association to synaptic vesicles and a modulatory role in the release of dopamine [20], although many questions remain over its function also.

The clinical use of levodopa in the treatment of PD motor dysfunction has changed little; patients take several doses over the course of the day, due to the drug’s short half-life [10]. As the disease progresses, the frequency of dosing increases, but eventually, possibly due to the continual loss of dopaminergic synapses in striatum, patients experience the phenomena of “wearing-off”, whereby motor symptoms appear at the end of each dosing window; this is a significant problem for patients [9]. In addition, dyskinesias (involuntary, writhing-like movements) are well known and are linked to the combination of PD with the long-term, pulsatile use of levodopa [9]. Data suggesting a more continuous plasma level is beneficial in reducing these motor complications have existed for many years [21], and a very recent, large Phase III trial proved this point [22].

Nevertheless, PD is not just a movement disorder. It has long been known that peripheral signs, such as constipation and olfactory impairment, are observed in patients many years prior to the diagnostic movement dysfunction [8]. Moreover, skin, the largest organ of the body, is now becoming a major player in PD. In a very large, recent population study, skin symptoms were found to rise in the years prior to diagnosis of PD [15]. The vast majority of patients show loss of intraepidermal nerve fibres ([23]; IENFs)—the small fibres in skin epidermis—and the loss of IENF progresses with disease [24,25]. The cell bodies of skin sensory (nociceptive) neurons reside in dorsal root ganglia, which carry Lewy bodies from early stages in PD [19]. Symptoms of polyneuropathy are highly prevalent in patients [26], and are seen at diagnosis [27] and prior to diagnosis of PD [28].

Importantly, sensory deficits impact motor function by affecting the patient’s ability to gauge balance [29] and indeed, the Unified PD rating scale correlates with IENF loss [30]. Certainly, issues with gait and balance are clearly identified as refractory to current treatments and are a matter for urgent therapeutic development [10]. Thus, patients show several ongoing changes in skin, changes in skin sensation appear prior to the motor signs that are used for diagnosis, and disease-induced impairment of sensory neurons may underlie important symptoms that are currently undertreated in the clinic. Moreover, skin measures may be able to quantitatively and objectively monitor disease, particularly as skin-punch biopsies are safe and have a very low prevalence of side effects [31].

However, high-dose levodopa itself may also contribute to the neuropathy that is observed in PD. In the clinic, high-dose levodopa has repeatedly been associated with exacerbation or development of neuropathy symptoms [10,23] and has even been shown to impair spinal conduction in as little as one month [32]. Moreover, loss of epidermal sensory neurites (IENF) correlates with oral levodopa dose [30].

Here, in this paper, we show for the first time that levodopa is deleterious to mitochondria, beta tubulin and lysosomes in primary cultures of dorsal root ganglia. We show that levodopa, at concentrations observed in patients, exacerbates mitochondrial toxicity induced by the pesticide rotenone, suggesting that, in vivo, levodopa may add to parkinsonian pathophysiology. Levodopa also reduced lysosome content in a sensory neuronal cell line. This effect was not prevented by entacapone. Thus, our translationally relevant data show that levodopa may contribute to peripheral neuropathy in PD patients.

## 2. Materials and Methods

### 2.1. Dorsal Root Ganglia

#### 2.1.1. Dorsal Root Ganglia Preparation

Sprague Dawley pups (P5-P14) were euthanised by decapitation and dorsal root ganglia (DRGs) isolated and placed in ice-cold sterile PBS. DRGs were transferred to a dissociation solution (2 mg/mL collagenase, + 0.1 mg/mL DNase; Gibco, Basel, Switzerland) and incubated for 40 min at 37 °C. Cells were then incubated in trypsin (0.05%) for an additional 5 min at 37 °C, then in DMEM/F-12 + 10% foetal bovine serum (SigmaAldrich, Taufkirchen, Germany) to quench trypsinization. Cells were then triturated and centrifuged (10 min at 600× *g*), and the resulting pellet was resuspended in fresh complete medium (Neurobasal-A (Gibco, Basel, Switzerland) supplemented with B-27 (Thermofisher, Basel Switzerland), gentamicin and glutamine (GlutaMAX, Thermofisher, Basel, Switzerland)). An equivalent of 2 ganglia per 50 µL were plated per quadrant of 4-quadrant dishes (Cell Vis 4-chamber glass-bottom dishes; IBL, Gerasdorf Austria). Coverslips were coated with laminin and poly-D-lysine. After 3–4 h at 37 °C and 5% CO_2_, cells were supplemented with complete medium (Neurobasal A (ThermoFisher, Basel, Switzerland), B27 supplement (ThermoFisher, Basel Switzerland), Gentamicin and GlutaMAX (Thermofisher, Basel, Switzerland)). The following day, the medium was replaced with fresh medium containing 1.5 µM cytarabine (Ara-c; SigmaAldrich, Taufkirchen, Germany) to reduce proliferation of fibroblasts.

#### 2.1.2. Dorsal Root Ganglia Treatments

**Figure 1:**
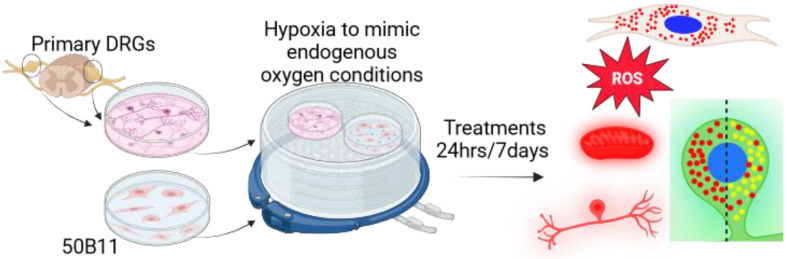
Primary cultures of DRGs were prepared and treated for 24 h or 7 days in hypoxia (hypoxia to mimic endogenous conditions and prevent levodopa auto-oxidation). Cultures were then examined for mitochondrial membrane potential (tetramethylrhodamine, methyl ester (TMRM)), reactive oxygen species (ROS) using dihydroethidium, beta III tubulin and lysosome content using Lysotracker red (red dots in the green DRG soma) and lysosome acidity (Lysotracker red + Lysosensor green, red + green = yellow dots in green DRG soma) as detailed in the text. Cells from the 50B11 cell line were also treated, then cultured in hypoxia for 24 h, and lysosome content was examined, as detailed in the text, using Lysotracker (red dots in the cell soma at top right). Figure made using BioRender.

At approximately DIV 7, cells were placed into a hypoxia chamber (hypoxia: 3% O_2_, 5% CO_2_ and 92% N_2_) for 3 days to habituate. After 3 days, the cells were treated with rotenone (SigmaAldrich, Taufkirchen, Germany; 0, 1 nM, 10 nM, 100 nM, 500 nM) and/or levodopa methyl ester (0, 3 µM, 30 µM, 300 µM) for 24 h or 7 days. Rotenone is widely used to model parkinsonism [17]. Hypoxia was used to prevent auto-oxidation of levodopa [33]. Please see Figure 1 for graphical explanation. Vehicle controls for rotenone and levodopa were DMSO (SigmaAldrich, Taufkirchen, Germany) and distilled water, respectively. For some oxidative stress experiments, additional cells were incubated in normal atmosphere (normoxia, 5% CO_2_, 20% O_2_) and were treated in parallel.

#### 2.1.3. Measurement of Mitochondrial Membrane Potential

Membrane potential was measured using TMRM (Thermofisher, Basel, Switzerland) in hypoxic conditions only. The medium was removed and replaced with medium containing 10 nM TMRM (non-quenching [34,35]) and treatments (see above) for 30 min. Cells were then imaged using a confocal microscope (Zeiss, Oberkochen, Germany; LSM780, ex 561 nm, em 566–669 nm; 0.05 × 0.05 µm per pixel, 53.14 × 53.14 µm total field of view taken using Plan-Apochromat 40×/1.3 oil DIC M27 objective). Laser settings were consistent within each experiment. The photomicrographs were analysed using ImageJ software (V1.54f). Briefly, each soma was outlined to create an ROI and the mean pixel intensity of TMRM-stained mitochondria measured. At 24 h: N = 3 experiments, N = 9–19 cells per condition per experiment, 32–41 cells per condition overall. At 7 days: N = 6 experiments, N = 4–60 cells per condition per experiment, N = 99–173 cells per condition overall.

#### 2.1.4. Oxidative Stress Assay

Dihydroethidium (DHE; Santa Cruz Biotechnology) was used to measure reactive oxygen species (ROS) in live cells. Briefly, DHE was added to cells (final concentration 10 µM DHE) that were then incubated for 30 min. Cells were then imaged using a confocal microscope (LSM780, ex 561 nm, em 585–733 nm; 0.83 × 0.83 µm per pixel, 1024 × 1024 pixels, taken using Plan-Apochromat 10×/0.45 M27 objective, Zeiss, Oberkochen, Germany). Subsequently, the images were analysed with ImageJ using particle analysis. Briefly, images were thresholded using manual intensity threshold boundaries of 120–255. Particle sizes 200 µm^2^–infinity in size and with a circularity of 0.10–1.00 were measured, which ensured that only DRG neurons were quantified and avoided the fibroblasts present in our mixed cultures (cultures were treated with Ara-c but were considered mixed as some fibroblasts remained) providing an outcome of percent area above threshold per image. Normoxia 24 h treatment: N = 1–8 images per condition per experiment, N = 4 experiments and N = 16–24 images per condition in total. Hypoxia 24 h treatment: N = 1–15 images per condition per experiment, N = 4 experiments and 13–30 images per condition in total. Hypoxia 7 days treatment: N = 3–40 images per condition per experiment, N = 5 experiments and 74–142 images per condition in total.

#### 2.1.5. Immunostaining

Cells were fixed for 10 min using 4% paraformaldehyde (SigmaAldrich, Taufkirchen, Germany) containing 250 mM sucrose (Fisher Scientific, Dreieich, Germany) and stored at −4 °C for subsequent immunostaining. Following washing (3 × 5 mins in 0.01 M PBS), cells were then permeabilised with 0.1% Triton X-100 for 10 min, then blocked in 5% goat serum (diluted in 0.01 M PBS: block, Jackson ImmunoResearch, Ely, UK) for 1 h. Cells were then incubated in primary antibodies, diluted in block, and left overnight with gentle rotation (anti-MAP2, Abcam cat# ab5392 RRID:AB_2138153, 1:10 000; anti-beta III tubulin antibody, Abcam Cat# ab18207, RRID:AB_444319, 1:2500; anti-ATP5b, Millipore Cat# MAB3494, RRID:AB_177597, 1:500). Cells were washed (3 × 5 mins in 0.01 M PBS), then incubated with secondary antibody for 2 h (Jackson ImmunoResearch, 1:200 in block). Following washing (3 × 5 mins in 0.01 M PBS), cells were counterstained with 0.5 μg/mL Hoechst solution (Hoechst-34580 dye, SigmaAldrich, Taufkirchen, Germany) for 10 min, then washed in PBS. Finally, a drop of fluorescence mounting medium was added to each quadrant, and dishes were then stored at −20 °C until imaging.

Neurites based upon MAP2 staining were imaged using LSM780 AxioObserver, plan-apochromat 10×/0.45 M27 objective, Zeiss, Oberkochen, Germany; image resolution 1.38 × 1.38 µm/pixel, 1024 × 1024 pixels/image. Excitations and emissions were 405 nm, 410–585 nm (Hoechst, SigmaAldrich, Taufkirchen, Germany), 488 nm, 490–594 nm (MAP2). A total of 4–13 images were taken per treatment per timepoint per experiment. Percent area positive for MAP2 staining was determined following auto-thresholding in ImageJ. Data were then expressed as percent of control-treated cells at each timepoint. There were 1–14 photomicrographs per condition per experiment, N = 4 experiments and 10–51 images per condition in total.

Neurites based upon beta III tubulin staining were imaged using LSM780 AxioObserver, plan-apochromat 10×/0.45 M27 objective, image resolution 1.66 × 1.66 µm/pixel, 512 × 512 pixels/image. Excitations and emissions were 405 nm, 410–499 nm (Hoechst), 561 nm, 585–733 nm (beta III tubulin). Channels were separated; the red channel was auto-thresholded, then percent area above threshold per image was quantified in ImageJ (Fiji, 1.54f [36]). Using the thresholded images to “IdentifyPrimaryObjects”, fluorescence intensity per neurite per image was quantified from the red channel (beta III tubulin) in CellProfiler (V2.4.6 [37]). There were 20–40 images per condition from N = 3 experiments.

Cell bodies were imaged using LSM780 AxioObserver, plan-apochromat 40/1.3 oil DIC M27 objective, image resolution 0.05 × 0.05 µm/pixel, 1024 × 1024 pixels/image. Excitations and emissions were 405 nm, 415–572 nm (Hoechst), 488 nm, 490–594 nm (ATP5b), 561 nm, 585–733 nm (beta III tubulin). The images were then analysed in ImageJ [36]. To determine mitochondrial content in soma, channels were split and each soma was outlined from beta III tubulin staining, avoiding the nucleus. Staining for ATP5b was auto-thresholded, and the percent area above threshold, within the soma ROIs, was quantified and expressed relative to control-treated cells. Control cells that were incubated with no primary antibody showed no staining. There were 5–66 cells per condition per experiment, N = 3 experiments and 130–131 cells per condition in total.

#### 2.1.6. Lysosome Content and Acidity Measurements

Following treatment with control or 300 µM levodopa in hypoxia, the medium was removed from cells and replaced with medium containing treatments and Lysotracker red (100 nM, L7528 Thermofisher, Basel, Switzerland) and Lysosensor green (1 µM, L7535, Thermofisher, Basel, Switzerland). Cells were incubated in the dark, at 37 °C, for 1 h, washed gently and imaged in warmed Krebs. A 10 µm stack through the middle of each DRG soma, identified based upon Lysosensor, was taken using an LSM780 confocal Plan-Apochromat 63×/1.4 Oil DIC M27, Zeiss, Oberkochen, Germany (pixel size 0.7 µm × 0.7 µm × 0.34 µm). Using a beam splitter, a line scan was taken, ex 405 nm, em 410–556 nm (Lysosensor), ex 561 nm, em 566–691 nm (LysoTracker). For analysis, channels were split, auto-thresholded and despeckled. A 5 µm block of soma was outlined and surrounding area cleared. For total lysosomal content, the area stained by Lysotracker per optical slice was quantified, expressed as a proportion of cell area and a mean per soma generated. For acidity, the proportion of Lysosensor contained with Lysotracker-stained ROIs per slice was quantified and a mean per soma generated. A total of 28–40 soma were analysed per condition per experiment, N = 3 experiments with 98–104 cells per condition in total.

### 2.2. 50B11 Cells

#### 2.2.1. Culturing

50B11 cells were cultured in neurobasal medium supplemented with 10% foetal bovine serum, 2% B-27, 20 mM D-glucose and 0.2 M L-glutamine (GlutaMAX). All media and supplements were from ThermoFisher, Basel, Switzerland. Cells were used at passages 5–9.

#### 2.2.2. 50B11 Treatments

Cells were plated in poly-D-lysine-coated 4-quadrant dishes (Cell Vis 4-chamber glass-bottom dishes; Gerasdorf Austria). The following day, they were placed in a hypoxic chamber (3% O_2_, 5% CO_2_ and 92% N_2_) for 24 h to habituate. Cells were then treated with combinations of levodopa methyl ester (a more water-soluble version of levodopa, SigmaAldrich, Taufkirchen, Germany; 30 µM, 300 µM), entacapone (1 µM [38]), homocysteine (20 µM [39,40]) or vehicle (ddH_2_O) for 24 h. Please see Figure 1 for graphical explanation.

#### 2.2.3. Lysosome Analysis

Following 24 h of treatment, the medium was replaced with medium containing treatments and Lysotracker red (100 nM, L7528 Thermofisher, Basel, Switzerland) and Hoechst-34580 dye (500 nM; SigmaAldrich, Taufkirchen, Germany) for 30 min. Cells were then imaged using StereoInvestigator (V5.00, MBF Bioscience, Williston, VT, USA) on a Zeiss Z1 microscope at ×63 magnification. Lysotracker red is a well-known dye that is ion-trapped in acidic environments. Images of nuclei and lysosomes were taken separately through the depth of each cell using consistent settings for exposure, gain and binning within each experiment. The image stacks were then rendered using Helicon Focus (V8.2.2, Kharkiv, Ukraine) and cropped to 1550 × 1180 pixels. Resultant images were then processed in CellProfiler (V4.2.6; [37]). The size of all lysosomes was quantified from 7–24 images per condition per experiment, N = 4 experiments and a final number of 43–77 images per condition in total (20275–37932 lysosomes in total per condition). In addition, Hoechst-stained nuclei were co-localised within these photomicrographs in CellProfiler, then expanded to create masks using boundaries of 10, 20, 30, 40, 50 and 60 pixels from original size. Lysosomes within each boundary were identified. To create total content per boundary per cell, the total area occupied by lysosomes per boundary per image was divided by the total area of nuclei per image. As above, 7–24 images per condition per experiment, N = 4 experiments and a final number of 43–72 images per condition (5 lysosome photomicrographs for the homocysteine condition were not used as nuclei photomicrographs were unavailable).

### 2.3. Statistics

All analyses were conducted blinded and were then unblinded for statistical comparisons and graphing, which was performed using GraphPad Prism V10.2.3. The threshold for significance was *p*-value < 0.05. Where variances were similar (based upon F tests), Student t tests were used to compare two independent groups and 1-way ANOVAs were used to compare between groups varying by 1 factor. Where data showed significantly different variances, based upon F tests, unpaired t tests with Welch’s correction were used to compare two independent groups, and Brown–Forsythe ANOVA tests were used to compare between several groups varying by 1 factor, followed by Dunnett’s T3 multiple comparisons test. Two-way ANOVAs (mixed-effects model) followed by appropriate post hoc tests were used to compare data where there were two factors; equal variances were not assumed and the Geisser–Greenhouse’s epsilon correction was used. For large datasets (lysosome sizes), MATLAB Version: 24.1.0.2644111 (R2024a) Update 4 [41] was used for 2-way ANOVA using the *p* = ANOVA2 function. All conditions must have the same group size to use this function: a random non-repeating selection of 20,000 lysosome sizes were taken from each condition using an Excel macro. Datasets on individual lysosome sizes were tested for normality using D’Agostino and Pearson and Kolmogorov–Smirnov tests. For Kruskal–Wallis 1-way ANOVA analysis of this dataset, the full dataset was used. For graphs, data are shown as scatter plots showing individual technical replicates with mean ± SEM or as box and whisker plots of technical replicates with 95% confidence intervals, with remaining datapoints, medians and means shown, as recommended [42,43,44]. In all cases, experimental means are superimposed for reference. Sample size calculations show that, for DRGs, N = 27 soma are required to detect a change of 30% in mitochondrial membrane potential from control; N = 8 images are required to detect a difference of 30% from controls for DHE; N = 31 soma for lysosome acidity measurements (in each case, 80% power, alpha = 0.05). For 50B11 cells, N = 39 cells are required to detect a 30% change from control for lysosome content (power = 80%, alpha = 0.05).

## 3. Results

### 3.1. Establishing a Parkinsonian Primary Sensory Neuronal Cell Model

**Figure 2:**
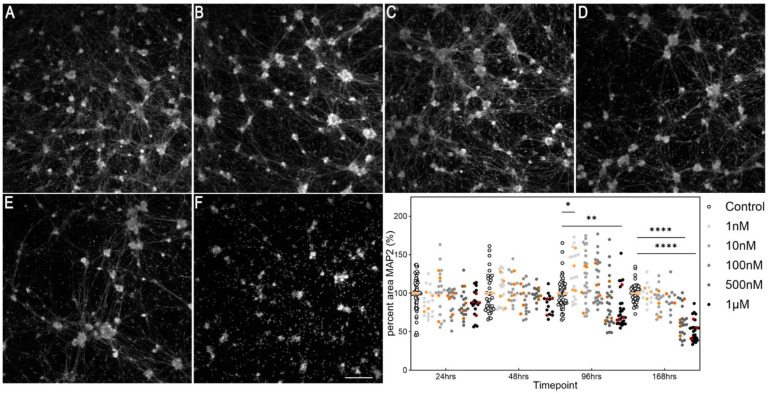
Time-dependent cytotoxicity of rotenone in DRGs. Cells were fixed and stained for MAP2, a pan-neuronal marker. (**A**–**F**) Example photomicrographs following 7 days (168 h) of treatment; (**A**) = control, (**B**) = 1 nM, (**C**) = 10 nM, (**D**) = 100 nM, (**E**) = 500 nM, (**F**) = 1 µM. Scale bar in (**F**) = 200 µm and is for all photomicrographs. Photomicrographs are not edited except for contrast, and they depict the MAP2 channel. The graph shows the time-dependent effect of rotenone on DRGs. Symbols in white and shades of grey to black show data from individual technical replicates. Symbols in shades of orange to brown show experimental means. * *p* < 0.05, ** *p* < 0.01, **** *p* < 0.0001.

We began our experiments with a preliminary study on the concentration of rotenone for inducing parkinsonism in primary cultures of dorsal root ganglia. Cultures were incubated in normoxia (21% O_2_) for these experiments. There was a consistent significant decline in MAP2 immunostaining by 4 days, at 1 µM rotenone, and by 7 days at 500 nM, primarily of neuronal processes (Figure 2; effect of treatment F (5, 178) = 21.3, *p* < 0.0001).

### 3.2. Effect of Levodopa on Sensory Neurons Alone and in the Context of Parkinsonism

#### 3.2.1. Levodopa Exacerbates Mitochondrial Impairment in Parkinsonism

Levodopa oxidises upon exposure to air [33], and in any case, the partial pressure of oxygen within the body is typically less than 5% [45]. Thus, culturing in a normoxic environment is actually hyperoxic with respect to endogenous conditions. Therefore, in order to examine the legitimate effects of levodopa on sensory neurons, we cultured in hypoxic conditions (3% O_2_). Although this is normoxic compared to within the body, for simplicity, we refer to 3% O_2_ as hypoxia/hypoxic. In vivo, in human patients, levodopa plasma levels fluctuate with each dose but concentrations vary from 1–10 mg/L [46,47,48,49,50,51]. These concentrations correlate to approximately 2–30 µM, and here, we treated with 3, 30 and 300 µM.

**Figure 3:**
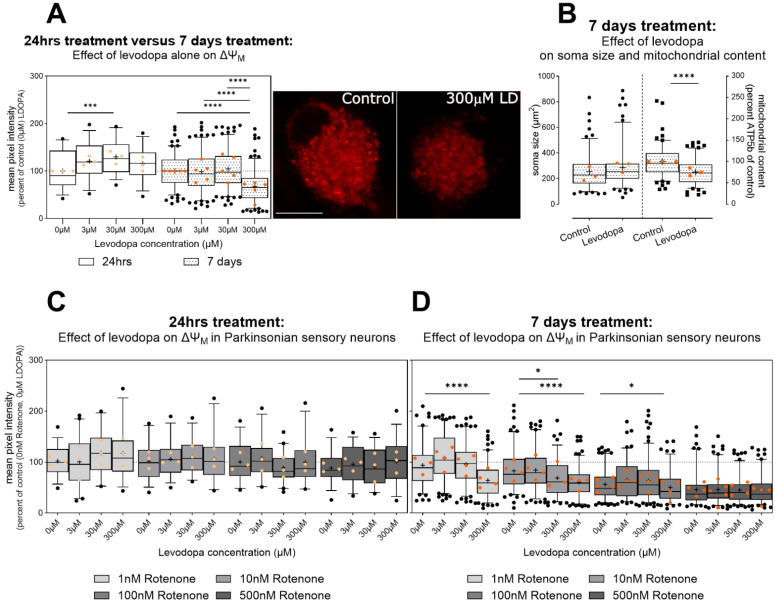
Effect of levodopa on mitochondrial membrane potential (ΔΨ_M_) in primary sensory neurons (DRGs) cultured in hypoxia. Data are normalized to percent of control cells treated with 0 µM levodopa and 0 nM rotenone in hypoxia. (**A**) A dose of 30 µM levodopa increases ΔΨ_M_ at 24 h in hypoxia; however, this effect is lost at 7 days and instead 300 µM levodopa inhibits ΔΨ_M_. Photomicrographs show TMRM staining in DRG soma treated with 0 µM levodopa (left) or 300 µM levodopa (LD, right) in hypoxia. Scale bar = 10 µm, for both images. (**B**) (left) Beta III tubulin immunocytochemistry shows no effect of high-dose (300 µM) levodopa on soma size; however, mean percent fluorescence for ATP5b (right), a mitochondrial marker, was reduced. (**C**) No impact of levodopa is observed in the context of parkinsonism (rotenone) at 24 h. (**D**) At 7 days, the deleterious effect of 300 µM levodopa is maintained in mild ΔΨ_M_ inhibition (1 nM rotenone). Further, both 30 µM and 300 µM levodopa reduce ΔΨ_M_ caused by 10 nM rotenone. No additive effects of levodopa are observed at stronger ΔΨ_M_ inhibition caused by 500 nM rotenone. Data in (**A**–**D**) are shown as box plots of technical replicates with whiskers depicting 5–95% percentiles, black circles depicting remaining data points, lines depicting medians and “+” symbols depicting means. Light and dark orange circles show experiment means. (**A**,**C**,**D**): dashed lines show 100% (mean of control cells not treated with levodopa or rotenone) for reference. * *p* < 0.05, *** *p* < 0.001, **** *p* < 0.0001.

Levodopa has previously been shown to impair mitochondrial function in mesencephalic cultures and cancer cell lines [33] in hypoxia. However, here, in primary sensory neurons, we found that after 24 h treatment, 30 µM levodopa increased ΔΨ_M_, suggesting hyperpolarization in hypoxic conditions (Figure 3A). By 7 days, this effect was lost and instead 300 µM levodopa reduced mitochondrial membrane potential by 32% (Figure 3A; effect of levodopa, F (2.91, 530.1) = 10.7, *p* < 0.0001, levodopa × time interaction F (3, 547) = 8.4, *p* < 0.0001).

There was no impact of levodopa in the context of parkinsonism (rotenone) at 24 h (Figure 3C, effect of levodopa F (2.5, 108.2) = 1.4, ns). However, high-dose levodopa maintained its detrimental effect on ΔΨ_M_ at 7 days in all rotenone conditions; indeed, 300 µM reduced ΔΨ_M_ to the same degree as 500 nM rotenone (Figure 3D). Moreover, 30 µM worsened ΔΨ_M_ in cells treated with 10 nM rotenone (Figure 3D, effect of levodopa F (2.8, 561.2) = 30.0, *p* < 0.0001; levodopa × rotenone interaction F (7.5, 514.2) = 7.9, *p* < 0.0001). This suggests that levodopa, at concentrations observed in patients, can exacerbate reductions in ΔΨ_M_. Moreover, high-dose levodopa, alone, impairs mitochondrial membrane potential in sensory neurons.

The loss in ΔΨ_M_ may be due to impaired electron transport impeding ATP production, as suggested by previous data at high-dose levodopa [33]; however, mitochondrial load itself may be reduced due to impaired transport [52]. We investigated this in a separate series of experiments where we analysed ATP5b content in DRG soma incubated for 7 days in hypoxia and treated with 300 µM levodopa or control. Although there was no change in soma size, based upon beta III tubulin, indeed, mean fluorescence for ATP5b was reduced, suggesting some loss of mitochondrial from DRG soma (area: Figure 3B left unpaired t test, t = 1.71, df = 254, ns; ATP5b fluorescence intensity Figure 3B right t test, t = 6.4 df = 254, *p* < 0.0001).

#### 3.2.2. Chronic Levodopa Initially Increases and Then Ameliorates Oxidative Stress, at Concentrations Observed In Vivo

Loss of mitochondrial membrane potential can lead to an increase in oxidative stress due to the failure of electron transport across complex I, in the case of rotenone, and subsequent transfer of the electron to acceptor molecules. We used dihydroethidium (DHE), which fluoresces when it reacts with reactive oxygen species.

After 24 h treatment with levodopa only, no oxidative stress was observed, in keeping with the lack of change in ΔΨ_M_ at this timepoint (Supplementary File S1, Figure S1A; no effect of levodopa F (2.2, 93.0) = 2.9, ns; levodopa × condition F (3, 127) = 1.4, ns). In parkinsonian (rotenone-treated) sensory neurons cultured in normoxia, the highest dose of levodopa tended to reduce oxidative stress at 300 µM (Supplementary File S1 Figure S1B: effect of levodopa F (2.5, 191.1) = 8.8, *p* < 0.0001, levodopa × rotenone interaction F (9, 228) = 4.1, *p* < 0.0001), in contrast to previous data [33]. In hypoxia, levodopa had no robust effects (Supplementary File S1 Figure S1C: effect of levodopa F (2.3, 176.3) = 4.6 *p* < 0.01, levodopa × rotenone interaction F (9, 227) = 2.2 *p* < 0.03). Thus, levodopa induced mild inconsistent effects on oxidative stress at 24 h in parkinsonian sensory neurons.

**Figure 4:**
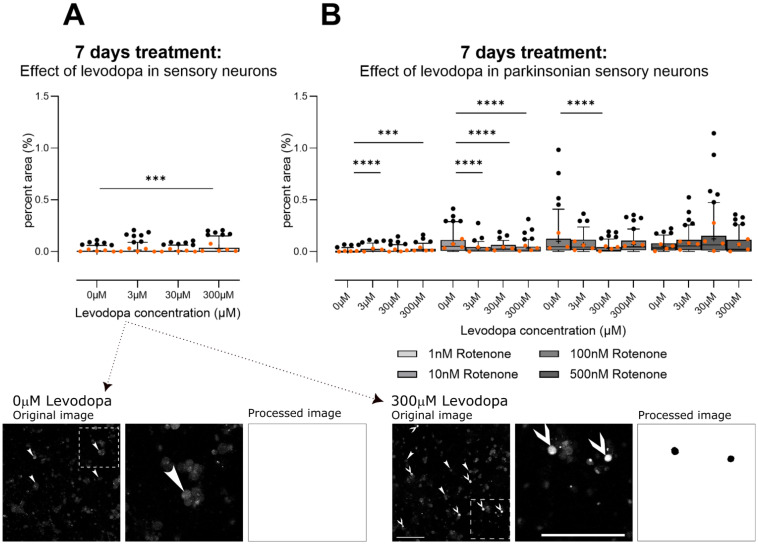
Effect of levodopa on oxidative stress in primary sensory neurons (DRGs) cultured in hypoxia. (**A**) Alone, only the highest concentration of levodopa (300 µM) induces mild oxidative stress. (**B**) In the context of parkinsonism (rotenone), levodopa increases oxidative stress at 1 nM rotenone. At moderate mitochondrial inhibition (10 nM, 100 nM), levodopa tended to reduce oxidative stress, possibly due to its ability to accept electrons [33] and this effect is lost at 500 nM rotenone. Data are technical replicates and are shown as box plots with whiskers depicting 5–95% percentiles, black circles depicting remaining data points, lines depicting medians and “+” symbols depicting means. Light and dark orange circles show means for each experiment. *** *p* < 0.001, **** *p* < 0.0001 post hoc tests, following ANOVA, as discussed in text. Photomicrograph for 0 µM levodopa shows example original image of cells treated with 0 µM levodopa for 7 days and stained using dihydroethidium, with outlined area shown zoomed in and its corresponding segmentation; no cells were positive for oxidative stress. Photomicrograph for 300 µM levodopa shows example original image of cells treated with 300 µM levodopa for 7 days, then stained with dihydroethidium, with outlined area shown zoomed in and its corresponding segmentation; two cells were positive for oxidative stress in the zoomed in area. Arrowheads in photomicrographs point to DRG soma negative for oxidative stress (e.g., 0 µM levodopa). Arrows in photomicrographs point to DRG soma positive for oxidative stress. Photomicrographs are not modified, except for cropping for the zoomed images. Image processing picked out only DRG soma that contained reactive oxygen species. Note that the segmentation was for the entire image; here, we have used zoomed in areas to better demonstrate the segmentation. Scale bars = 200 µm.

After 7 days of treatment in hypoxia, levodopa alone caused a mild increase in oxidative stress, correlating with the loss in ΔΨ_M_ at this levodopa concentration and timepoint (Figure 4A; Brown–Forsythe ANOVA test, F (3.0, 371.7) = 9.1, *p* < 0.0001). The lowest level of rotenone (1 nM) did not change ΔΨ_M_ and, in this context, levodopa also increased oxidative stress. However, at moderate levels of rotenone-induced inhibition of ΔΨ_M_ (10 nM, 100 nM), levodopa tended to reduce oxidative stress; this is not unexpected as the catechol group in levodopa may accept electrons [33] (Figure 4B: levodopa × rotenone interaction F (9, 1027) = 12.26, *p* < 0.0001). However, this ability may be overwhelmed at high levels of mitochondrial rotenone-mediated inhibition (Figure 4B: 500 nM).

#### 3.2.3. Levodopa Stabilizes Tubulin at Concentrations Observed In Vivo

**Figure 5:**
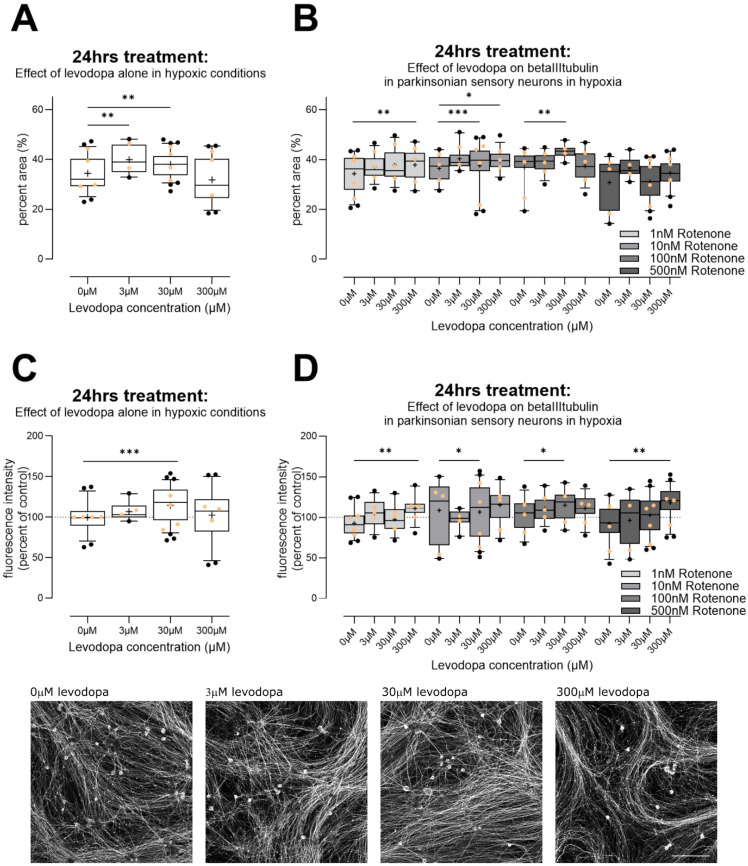
Effect of levodopa on beta III tubulin in primary sensory neurons (DRGs) cultured in hypoxia. (**A**) Alone, 3µM and 30 µM levodopa increased percent area immunoreactive for beta III tubulin. (**B**) In the context of parkinsonian sensory neurons (treated with rotenone), levodopa tended to increase percent area positive for beta III tubulin at 30 µM and 300 µM. (**C**) We examined fluorescence intensity for beta III tubulin, at the level of individual neurites in cells. Cells treated with levodopa only again showed increased fluorescence at 30 µM. (**D**) In the context of parkinsonian sensory neurons (treated with rotenone), 30 µM and 300 µM levodopa tended to increase fluorescence for beta III tubulin. Data are technical replicates shown as box plots with whiskers depicting 5–95% percentiles, black circles depicting remaining data points, lines depicting medians and “+” symbols depicting means. Light orange circles depict experimental means. * *p* < 0.05, ** *p* < 0.01, *** *p* < 0.001 post hoc tests, following ANOVA, as discussed in text. Photomicrographs show example original images of cells treated for 24 h in hypoxia with 0 µM, 3 µM, 30 µM, 300 µM levodopa, only, then stained for beta III tubulin. Photomicrographs are not modified. Scale bar bottom right = 200 µm, for all images.

Interestingly, levodopa has been suggested to incorporate into tubulin as a false amino acid and stabilize it, thereby preventing organelle transport [52] or interfering with protein degradation [53]. We therefore examined immunoreactivity for beta III tubulin, which is only found in neurons. Following 24 h in hypoxia, both 3 µM and 30 µM levodopa led to an increase in percent area immunoreactive for beta III tubulin, with 300 µM having no effect (Figure 5A, Brown–Forsythe ANOVA test F (3.0, 134.9) = 12, *p* < 0.0001). In the context of parkinsonism, i.e., rotenone treatment, levodopa similarly led to increased immunoreactivity, particularly at 30 µM, although 300 µM increased immunoreactivity at 1 nM and 10 nM rotenone (Figure 5B; effect of levodopa F (2.4, 266.3) = 15.8, *p* < 0.0001; levodopa × rotenone interaction F (9, 333) = 2.3, *p* < 0.02). We then examined fluorescence at the level of individual neurites. Again, 30 µM levodopa led to an increase in fluorescence in the context of levodopa alone (Figure 5C, Brown–Forsythe ANOVA test, F (3.0, 135.7) = 6.6, *p* < 0.001). In the context of parkinsonism, there was a trend towards dose-dependent increase in fluorescence, with 30 µM and 300 µM increasing fluorescence significantly (Figure 5D, effect of levodopa, F (2.5, 281.3) = 13.5, *p* < 0.0001, levodopa × rotenone interaction F (9, 333) = 2.4, *p* < 0.02).

#### 3.2.4. Levodopa Reduces Lysosome Content at Concentrations Observed In Vivo

Having established that 30 µM levodopa exacerbates ΔΨ_M_ loss and may stabilize beta III tubulin in the context of parkinsonism, we then examined lysosomes because mitochondrial impairment plays a strong role in inducing lysosome biogenesis [54] and microtubules are critical for lysosome motility [55], but also because impairment of lysosome function is strongly implicated in PD [56]. For these experiments, we examined effects of levodopa alone, in hypoxic conditions.

**Figure 6:**
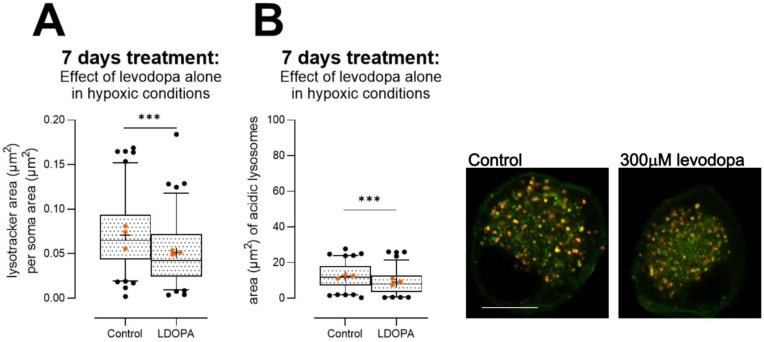
Effect of levodopa alone on lysosome content and acidity in primary DRGs cultured in hypoxia for 7 days. (**A**) A dose of 300 µM levodopa reduced lysosome content in DRG soma. (**B**) Acidity of lysosomes was impaired by 300 µM levodopa, because less lysosomes labelled with Lysotracker were co-labelled with Lysosensor. Data are shown as box plots with whiskers depicting 5–95% percentiles, black circles depicting remaining data points, lines depicting medians and “+” symbols depicting means. *** *p* < 0.001. Orange circles show experiment means. Photomicrographs depict control (left) and levodopa-treated (right) soma. Lysosomes were identified as Lysotracker (red)-positive puncta, and Lysosensor (green) was used to show appropriate acidity; thus, properly acidified lysosomes show co-localised (yellow) puncta. Scale bar = 10 µm, for both images.

In primary DRGs, despite the reduced mitochondrial membrane potential observed in DRGs at high-dose levodopa (Figure 3A), we observed reduced lysosomal content (Figure 6A, unpaired t test, t = 3.8, df = 200, *p* < 0.001), and a reduction in the proportion of lysosomes that were acidic (Figure 6B, unpaired t test t = 3.5, df = 200, *p* < 0.001).

We investigated these findings further in the 50B11 immortalized sensory neuronal cell line [57]. We examined in combination entacapone (1 µM, [38]), which is used as an inhibitor of catechol-o-methyl-transferase in patients to prevent metabolism of levodopa to 3-o-methyldopa and subsequent formation of homocysteine. This enzyme is expressed by 50B11 cells [58]. We also examined homocysteine itself (20 µM), used at concentrations that are observed in patients (10–20 µM [39,40]).

We first examined sizes of all lysosomes. As this resulted in very large group sizes, we extracted 20,000 lysosomes at random from the full dataset for each condition, to ensure an equal number for comparisons for the ANOVA. Both levodopa and entacapone affected lysosome size overall (effect of levodopa 2-way ANOVA, F (2, 119,994) = 119.43, *p* = 1.5 × 10^−52^; effect of entacapone F (1, 119,994) = 29.38, *p* = 5.9 × 10^−8^) and there was a strong interaction (F (2, 119,994) = 16.1, *p* = 1 × 10^−7^). We then analysed the complete datasets using non-parametric one-way analyses. A dose of 30 µM levodopa, alone, caused a mild 3% increase in median size of lysosomes. However, 300 µM levodopa had a strong effect and median size was reduced by 11% (Table 1, Kruskal–Wallis ANOVA H (3) = 175, *p* < 0.0001). Treatment with entacapone exacerbated these effects with 30 µM levodopa now causing a 6% loss in size and 300 µM resulting in a 14% loss in median size compared with cells treated with entacapone only (Table 1, Kruskal–Wallis ANOVA H (3) = 419.3, *p* < 0.0001). Homocysteine increased median lysosome size (homocysteine versus control-treated cells; Table 1, Mann–Whitney U = 449,847,789, *p* < 0.0001). Entacapone also showed no effect (control versus control + entacapone Mann–Whitney U = 437,670,689, ns). Lysosome sizes per experiment (median ± 95% CIs) are shown in Table S1. Thus, lysosome content is reduced by levodopa at concentrations observed in the patient.

As Lysotracker is sequestered in acidic environs, we also examined mean fluorescence of lysosomes. We observed very mild effects. At 30 µM levodopa, median fluorescence was increased by 2% from control-treated cells and at 300 µM levodopa, median fluorescence was reduced by 5% (Table S2; Kruskal–Wallis ANOVA H (3) = 1879, *p* < 0.0001). In the context of entacapone, median fluorescence was not changed by 30 µM levodopa but was reduced by 3% by 300 µM levodopa (Table S2; Kruskal–Wallis ANOVA H (3) = 614.0, *p* < 0.0001). Homocysteine caused a mild increase in fluorescence (compared with control-treated cells, Mann–Whitney U = 456,646,454, *p* < 0.0001), as did entacapone (compared with control-treated cells, Mann–Whitney U = 422,366,710, *p* < 0.0001). Thus, together, our data in 50B11 cells may reflect an absolute loss of lysosomes or a loss of acidic lysosomes. However, we note that our data in primary DRG soma (Figure 6) showed both a loss in lysosomes and remaining lysosomes were less acidic, thus it is likely that the same is occurring in 50B11 cells. Importantly, we show a loss of lysosomes at concentrations of levodopa and entacapone observed in the patient.

**Figure 7:**
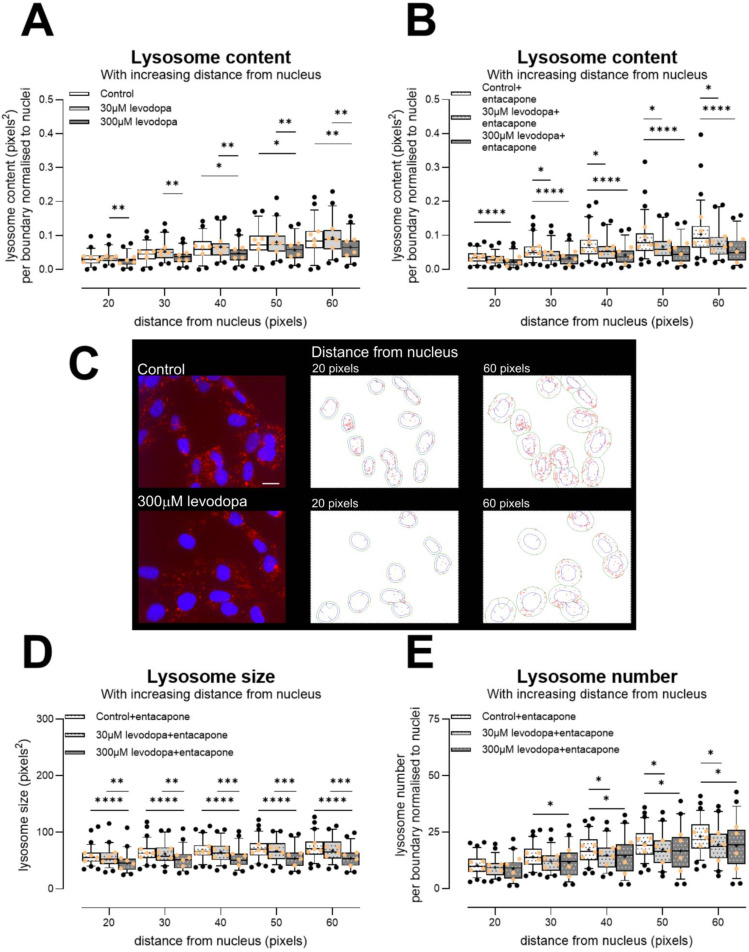
Effect of levodopa on lysosomes in the 50B11 immortalised sensory cell line, treated and incubated in hypoxia for 24 h. (**A**) A dose of 300 µM levodopa reduced lysosome content in comparison to control-treated cells and cells treated with 30 µM levodopa. (**B**) Both 30 µM and 300 µM levodopa reduced lysosome content when cells are treated with entacapone. (**C**) Photomicrographs show example images of cells treated with 0 µM or 300 µM levodopa for 24 h in hypoxia and labelled with Lysotracker (red) and Hoechst (nuclei). Photomicrographs are not modified except for brightness and contrast. Scale bar = 20 µm, for both photomicrographs. Outlines show examples of CellProfiler segmentations of lysosomes (red) at 20 pixels and 60 pixels from individual nuclei (blue). Note: For segmentation, the entire nucleus was required to be within the field of view, and not to touch the image border. The green lines show the appropriate boundaries. (**D**) Individual lysosome size per boundary, showing that in the context of entacapone, 300 µM levodopa in particular reduces lysosome size. (**E**) Number of lysosomes per boundary. In the context of entacapone, both 30 µM and 300 µM levodopa reduce lysosome number. Data in graphs are shown as box plots with whiskers depicting 5–95% percentiles, black circles depicting remaining data points, lines depicting medians and “+” symbols depicting means. * *p* < 0.05, **p < 0.01, ***p < 0.001, ****p < 0.0001. Light orange circles show experiment means. Entacapone = 1 µM [38].

These analyses are on the basis of individual lysosome size, and had a very large N, which may have contributed to the very robust statistical differences that we observed. We then moved to a separate and more conservative per-cell-based analysis by examining lysosome content at increasing distances from nuclei. As shown in Figure 7, we indeed observed a large reduction in total lysosome content at 300 µM levodopa irrespective of entacapone (Figure 7A, effect of levodopa F (2, 146) = 5.8, *p* < 0.01, distance × treatment F (8, 584) = 5.2, *p* < 0.0001; Figure 7B, effect of levodopa, F (2, 179) = 13.08, *p* < 0.0001, distance × treatment F (8, 716) = 11.8, *p* < 0.0001). Critically, the addition of entacapone to protect levodopa resulted in a reduction in lysosome content caused by 30 µM levodopa (Figure 7B). We also analysed these data using linear regression, and indeed, in the context of entacapone, the slope of increase in lysosome content within increasing distance from nucleus was reduced by both 30 µM levodopa (F (1, 621) = 6.1, *p* < 0.02) and 300 µM levodopa (F (1, 636) = 15.4, *p* < 0.0001).

With these smaller datasets, individual lysosome size was reduced in particular by 300 µM levodopa, in the context of entacapone (Figure 7D; F (2, 179) = 15.0, *p* < 0.0001, distance × treatment F (8, 716) = 2.7, *p* < 0.01). Moreover, the number of lysosomes was reduced by both 30 µM and 300 µM levodopa, in the context of entacapone (Figure 7E, F (2, 179) = 4.5, *p* < 0.05, distance × treatment F (8, 716) = 5.4, *p* < 0.0001). When we compared control-treated cells to homocysteine-treated cells, there was slightly greater content (Figure S2A) and lysosome number (Figure S2C). In these smaller datasets, there was no change in size caused by homocysteine (Figure S2B). These data again suggest that metabolism to homocysteine does not underlie the deleterious effects of levodopa. Entacapone itself had little effect (control versus control + entacapone lysosome content effect of treatment F (1, 126) = 2.3, ns; control versus control + entacapone lysosome size effect of treatment F (1, 125) = 1.2, ns). Thus, our data show in two separate cell types that even when using conservative analyses, concentrations of levodopa observed in the patient reduce lysosome content and number.

## 4. Discussion

Levodopa is undoubtedly an important drug; it is on the WHO list of essential medicines [59] and is preferred among patients and neurologists for the treatment of the bradykinesia of PD [7,8,9,10]. However, at high doses it has been shown to exacerbate or initiate symptoms of peripheral neuropathy in patients [10,23]. It has been shown to impair nerve conduction in as little as one month in humans with effects worsening at later timepoints [32]. Additionally, oral levodopa dose correlates with loss of intraepidermal nerve fibres (IENFs) in skin [30].

With regard to the mechanism underlying these effects, levodopa is metabolized by the enzymes dopa decarboxylase and catechol-o-methyl transferase (COMT) [60]. The conversion by COMT leads to the formation of 3-o-methyldopa and homocysteine, both of which form reactive oxygen species and are toxic in vivo and in vitro [61,62,63]. Increased levels of homocysteine have repeatedly been found in PD [64,65,66,67] and are associated with cognitive decline in PD [65,67]. However, whether homocysteine plays a role in sensory deficits is unknown. Vitamin B12 is a co-factor in the catabolism of homocysteine and recommendations have been made that PD patients are treated with B vitamins including B12 and B6 to prevent peripheral neuropathy [10]. Other mechanisms have also been postulated: high-dose levodopa has been linked to reduced mitochondrial respiration in immortalised mesencephalic cells and cancer cell lines [33,68]. Moreover, levodopa is incorporated as a false amino acid into proteins, which inhibits proteolysis [53]. Incorporation of the false amino acid into tubulin may interfere with organelle transport [52].

Here, we indeed noted depolarization of ΔΨ_M_ at high chronic concentrations (300 µM) in primary sensory neurons, which is in keeping with these previous data on high-dose levodopa in cancer cell lines [33]. Extending previous data that examined short timepoints [33], the impairment in ΔΨ_M_ caused by high-dose levodopa alone was associated with increased oxidative stress.

We further showed that levodopa causes hyperpolarization of the mitochondrial membrane potential (ΔΨ_M_) at low doses (30 µM). However, in parkinsonian sensory neurons treated chronically with rotenone, low-dose levodopa exacerbated loss of ΔΨ_M_, suggesting that even low-dose levodopa can impair mitochondrial function in conditions of pre-existing mitochondrial dysfunction. We note that our low-dose levodopa mimics concentrations observed in the patient [46,47,48,49,50,51]. In the context of parkinsonism, low-dose levodopa reduced oxidative stress, presumably due to the limited ability of the levodopa molecule to scavenge free radicals [33]. Again at concentrations observed in the patient, levodopa stabilized beta III tubulin content over the short term, an important finding as previous data suggested that very high concentrations were required for this outcome [52].

Finally, and critically, despite the impairment of mitochondria at high doses, which would be expected to enhance lysosome biogenesis [54], we show that high-dose levodopa leads to *reduced* lysosomal content, and reduced acidic lysosomes in primary sensory neurons. These results were reproduced in 50B11 cells, which are immortalized sensory neurons [57], where individual lysosome size was reduced per cell at the highest concentration of levodopa. Moreover, when we co-treated our cells with low-dose levodopa and entacapone to protect levodopa from metabolism, effects on lysosomes were additive in 50B11 cells, with low-dose levodopa reducing content and number of lysosomes. Interestingly, homocysteine tended to have the opposite effect, causing a weak increase in content and number. These data suggest again that levodopa underlies the deleterious effects rather than homocysteine. Entacapone is an inhibitor of the enzyme COMT, which metabolises levodopa and leads to the formation of homocysteine, and that COMT is expressed by 50B11 cells [58]. Entacapone had very little effect on lysosomes.

Lysosome number and size are constantly changing within the cell as a homeostatic response to, e.g., nutrient availability. During nutrient deprivation and induction of autophagy, lysosome size typically increases and number decreases [69], and they also tend to sequester to the perinuclear area [70]. In lysosomal storage diseases, the inability to degrade substrates causes a massive increase in lysosomes size, and lysosomes are also increased in size in response to TMEM106B overexpression [69]. Lysosomes may act as energy sensors; thus, it is possible that inhibitory effects of levodopa at the mitochondria have knock-on effects on the lysosome. Indeed, loss of the V-ATPase decreases lysosome number and size [69,70], and this proton pump is critical for lysosome acidification and its activity is dependent upon energy levels [71].

Given the strong impact of translationally relevant concentrations of levodopa on tubulin that we observed here, and that previously very high levels of levodopa have stabilized tubulin and impaired mitochondrial transport [52,72], it is possible that lysosome content was impacted by a reduction in motility. However, this is unlikely to underlie the loss in acidity that we observed. Our data clearly show that already at concentrations observed in the patient, levodopa is deleterious towards lysosomes. We note that this low concentration did reduce oxidative stress in parkinsonian sensory neurons and stabilized tubulin over the short term; however, we do not maintain these as beneficial effects; the same concentration also impaired ΔΨ_M_ in parkinsonian sensory neurons.

Impairment of lysosome acidification can lead to inflammation and cell death in vitro and may contribute to pathogenesis in several neurodegenerative diseases [73]. Lysosomes are well established as being impaired in PD; lysosomes are the main organelle that degrades alpha synuclein [18]. Mutations in *GBA1* and *LRRK2* each increase the risk of PD and both GBA1 and *LRRK2* are important for lysosomal function as GBA1 encodes the lysosomal enzyme β-glucocerebrosidase [18] and LRRK2 may control lysosome levels within the cell [74]. Most PD patients are treated with levodopa and for very good reasons [7,8,9,10]. However, our data suggest that levodopa may contribute to peripheral neuronal dysfunction and may exacerbate lysosomal and mitochondrial impairment. Moreover, the lysosome releases vitamin B6 and B12 from their binding proteins [75,76], suggesting that levodopa may inhibit the desired effect of free B vitamins in patients.

## 5. Conclusions

Levodopa, a critical drug for the treatment of PD, may be metabolized to homocysteine, which is toxic and thought to underlie levodopa-induced neuropathy. As vitamin B12 contributes to homocysteine catabolism, calls have been made to treat PD patients with B12 [77] to alleviate levodopa-mediated neuropathy. Here, in a translationally relevant series of experiments in sensory neurons, we showed that at concentrations observed in the patient, levodopa exacerbated mitochondrial impairment in parkinsonian cells, stabilized tubulin in parkinsonian cells and reduced lysosome content and acidity. In contrast, homocysteine had very little effect on lysosome content and may even increase content. Given the importance of lysosomes for release of B12 into the cell, and that low levels of B12 are a risk factor for peripheral neuropathy, our data suggest that B12 supplements may not be sufficient to protect against levodopa-induced neuropathy or that higher doses may be required.

## Figures and Tables

**Table 1:** Population estimates of median individual lysosome size ^1^ in 50B11 cells treated for 24 h in hypoxia.

Condition	Median (95% CIs ^2^)	Dunn’s Multiple Comparisons Test (Versus Control)	Dunn’s Multiple Comparisons Test (Versus Control + Entacapone)	Mann–Whitney U Test Compared with Control Cells (No Entacapone)
Control	35 (34–35)	-		
30 µM levodopa	36 (35–36)	*		
300 µM levodopa	31 (30–32)	****		
Control + 1 µM entacapone	35 (35–36)		-	
30 µM levodopa + 1 µM entacapone	33 (32–33)		****	
300 µM levodopa + 1 µM entacapone	30 (29–30)		****	
Homocysteine 20 µM	38 (37–38)			****

^1^ In pixels, as is standard for CellProfiler output. ^2^ 95% confidence intervals of median * *p* < 0.05, **** *p* < 0.0001.

## Data Availability

Research data are available at https://doi.org/10.23673/re-477.

## Supplementary Materials

The following supporting information can be downloaded at: https://www.mdpi.com/article/10.3390/biology13110893/s1, Figure S1: Effect of levodopa on oxidative stress in primary sensory neurons (DRGs) cultured in normoxia or hypoxia for 24 h. (A) Cells treated with levodopa alone for 24 h show no oxidative stress whether in normoxia or hypoxia. (B) In the context of parkinsonian (rotenone-treated) sensory neurons, 300 μM levodopa tended to reduce oxidative stress in normoxia. (C) Levodopa had no consistent effects in hypoxia. Data are technical replicates shown as box plots with whiskers depicting 5–95% percentiles, symbols depicting remaining data points, lines depicting medians and “+” symbols depicting means for optimal interpretation [42,43,44]. Light and dark orange symbols show experimental means (N = 4 for normoxia and for hypoxia). * *p* < 0.05, ** *p* < 0.01; Figure S2: Lysosome content ((A), effect of treatment F (1, 127) = 6.7, *p* < 0.04) and number ((C), effect of treatment F (1, 127) = 10.3, *p* < 0.01) is increased slightly by 20 μM homocysteine after 24 h treatment in hypoxic conditions. There is no effect on individual lysosome size ((B), effect of treatment F (1, 126) = 2.1, ns) in this small dataset. 50B11 cells (immortalised sensory neuronal cell line [57]) were habituated to hypoxic conditions and then treated and incubated for 24 h in hypoxia. Data are shown as box plots with whiskers depicting 5–95% percentiles, black circles depicting remaining data points, lines depicting medians and “+” symbols depicting means for optimal interpretation [42,43,44]. Experiment means are superimposed as light-orange circles. * *p* < 0.05, ** *p* < 0.01, *** *p* < 0.001; Table S1: 95% confidence intervals of median lysosome sizes per experiment. Individual lysosome sizes (standardized, in pixels) in 50B11 cells treated for 24 h in hypoxia; Table S2: Median fluorescence of individual lysosomes in 50B11 cells treated for 24 h in hypoxia *.
