# Supplementary material for: Levodopa Impairs Lysosomal Function in Sensory Neurons In Vitro
Source: Biology (Basel). 2024 Nov 2;13(11):893. doi: 10.3390/biology13110893 (PMC11591693; doi:10.3390/biology13110893)
Supplement: Supplementary file 1 [file biology-13-00893-s001.zip › biology-3243974-supplementary.pdf]

Supplementary file S1.

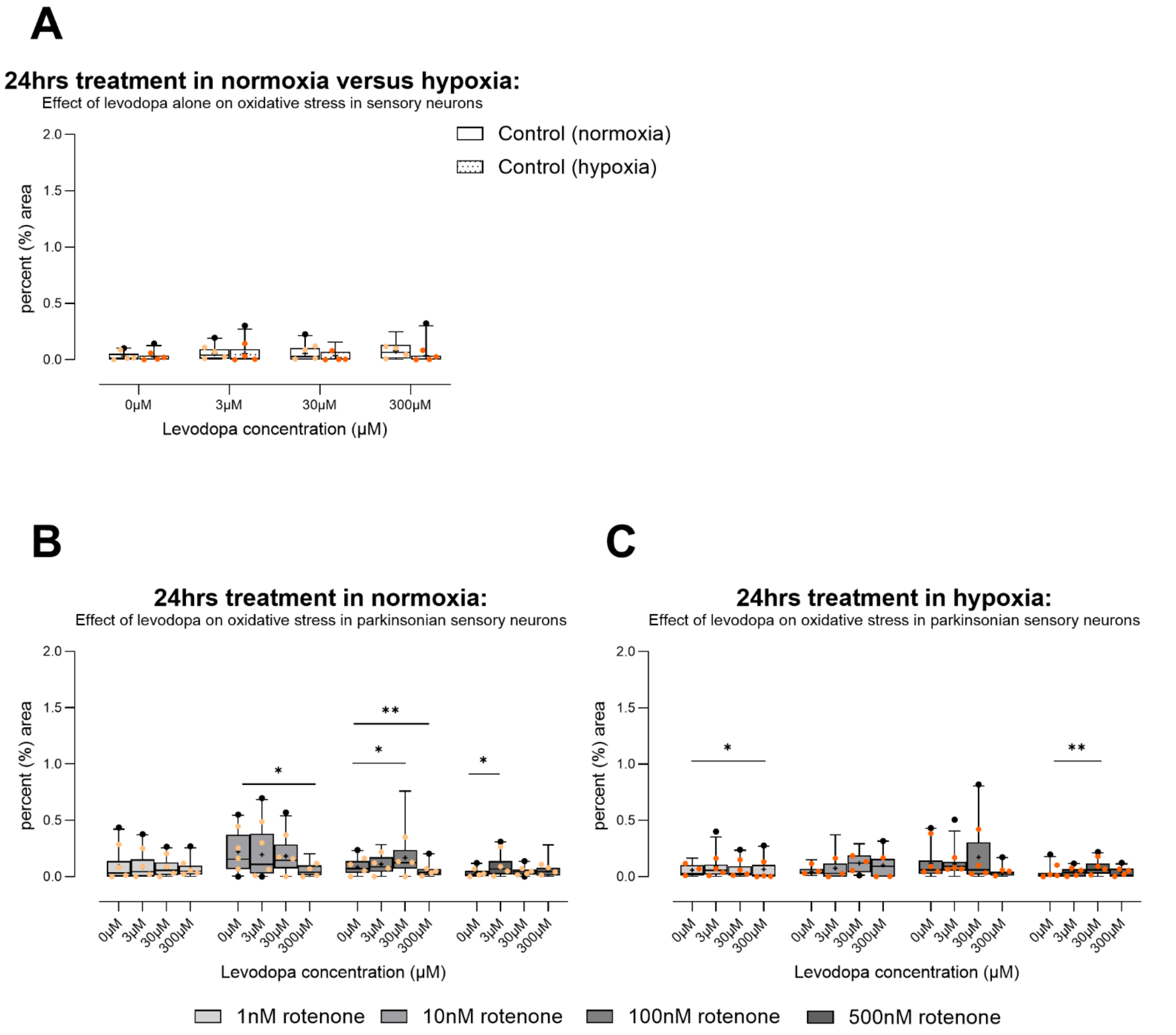

Figure S1 Effect of levodopa on oxidative stress in primary sensory neurons (DRGs) cultured in normoxia or hypoxia for 24 hours. A) Cells treated with levodopa alone for 24hrs show no oxidative stress whether in normoxia or hypoxia. B) In the context of parkinsonian (rotenone-treated) sensory neurons, 300μM levodopa tended to reduce oxidative stress in normoxia. C) Levodopa had no consistent effects in hypoxia. Data are technical replicates shown as box plots with whiskers depicting 5-95% percentiles, symbols depicting remaining data points, lines depicting medians and "+" symbols depicting means for optimal interpretation[1–3]. Light and dark orange symbols show experimental means (N=4 for normoxia and for hypoxia). *p<0.05, **p<0.01.

Table S1 95% confidence intervals of median lysosome sizes per experiment. Individual lysosome sizes (standardized, in pixels) in 50B11 cells treated for 24hrs in hypoxia.

Biologic replicate	Control	Control + 1µM entacapone	30µM levodopa	30µM levodopa + 1µM entacapone	300µM levodopa	300µM levodopa + 1µM entacapone	Homocysteine 20µM
1	33-36	34-37	30-33	27-29	24-26	21-23	35-38
2	33-36	32-34	35-38	33-35	33-35	30-31	36-37
3	35-37	38-40	36-38	36-38	31-33	32-33	41-44
4	32-34	31-34	34-36	29-31	28-31	27-29	35-36

Table S2 Median fluorescence of individual lysosomes in 50B11 cells treated for 24hrs in hypoxia*.

Condition	Control<br>Median (95% CI of median)	With entacapone<br>Median (95% CI of median)
Control	0.95 (0.95-0.96)	0.95 (0.95-0.95)
30µM LDOPA	0.97 (0.97-0.98)	0.95 (0.95-0.95)
300µM LDOPA	0.90 (0.90-0.91)	0.92 (0.92-0.92)
Homocysteine	0.96 (0.95-0.96)	-

*Fluorescence is normalised to mean of control-treated cells.

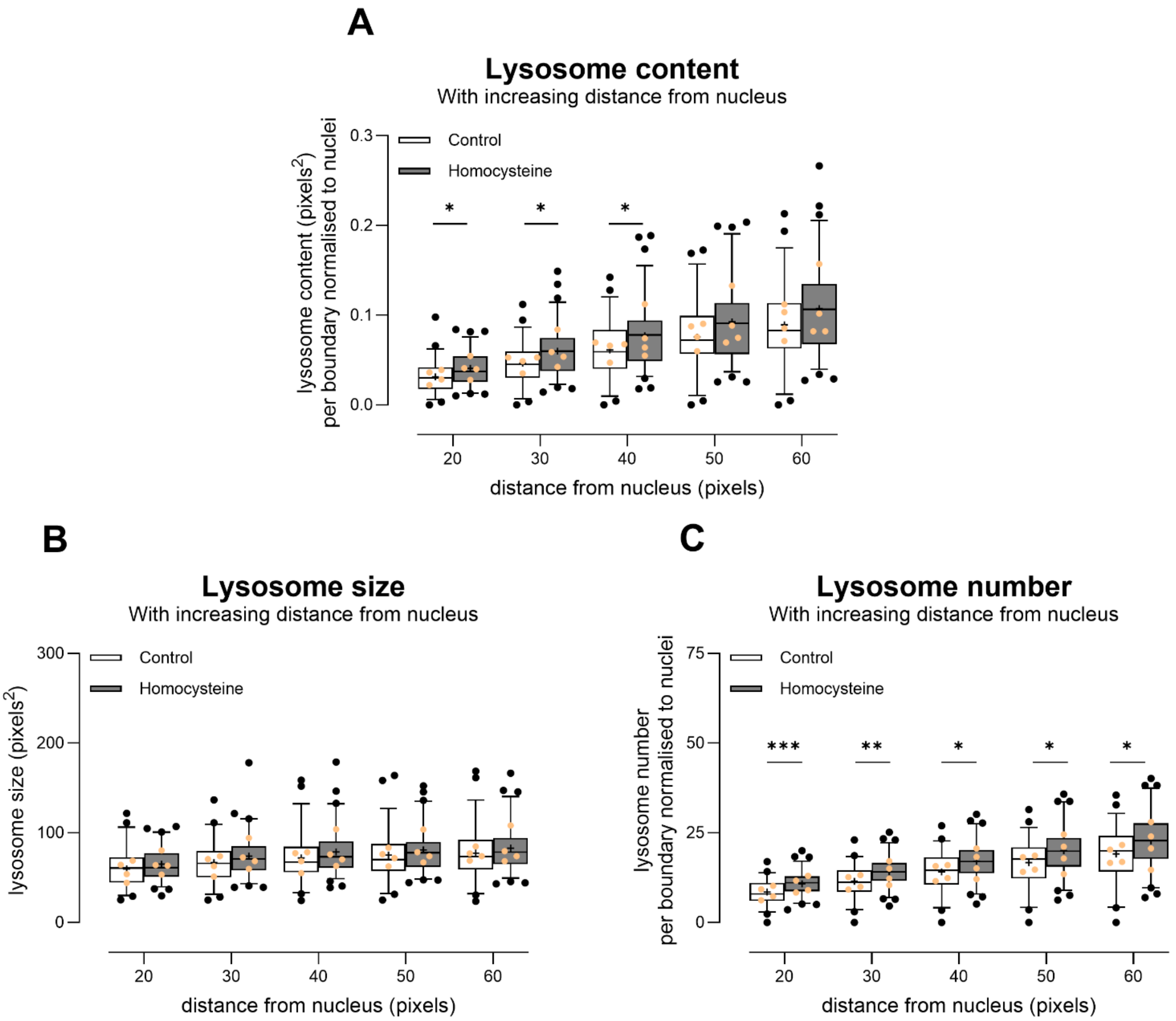

Figure S2 Lysosome content (A, effect of treatment F (1, 127) = 6.7, p<0.04) and number (C, effect of treatment F (1, 127) = 10.3, p<0.01) is increased slightly by 20µM homocysteine after 24hrs treatment in hypoxic conditions. There is no effect on individual lysosome size (B, effect of treatment F (1, 126) = 2.1, ns) in this small dataset. 50B11 cells (immortalised sensory neuronal cell line[4]) were habituated to hypoxic conditions and then treated and incubated for 24hrs in hypoxia. Data are shown as box plots with whiskers depicting 5-95% percentiles, black circles depicting remaining data points, lines depicting medians and "+" symbols depicting means for optimal interpretation[1–3]. Experiment means are superimposed as light-orange circles. * p<0.05, **p<0.01, ***p<0.001.

Bibliography

1. Heckman, M.G.; Davis, J.M.; Crowson, C.S. Post Hoc Power Calculations: An Inappropriate Method for Interpreting the Findings of a Research Study. *J Rheumatol* **2022**, *49*, 867–870, doi:10.3899/jrheum.211115.
2. Dziak, J.J.; Dierker, L.C.; Abar, B. The Interpretation of Statistical Power after the Data Have Been Gathered. *Curr Psychol* **2020**, *39*, 870–877, doi:10.1007/s12144-018-0018-1.
3. Greenland, S.; Senn, S.J.; Rothman, K.J.; Carlin, J.B.; Poole, C.; Goodman, S.N.; Altman, D.G. Statistical Tests, P Values, Confidence Intervals, and Power: A Guide to Misinterpretations. *Eur J Epidemiol* **2016**, *31*, 337–350, doi:10.1007/s10654-016-0149-3.
4. Chen, W.; Mi, R.; Haughey, N.; Oz, M.; Höke, A. Immortalization and Characterization of a Nociceptive Dorsal Root Ganglion Sensory Neuronal Line. *Journal of the Peripheral Nervous System* **2007**, *12*, 121–130, doi:10.1111/j.1529-8027.2007.00131.x.
